# Impact of the Ebola virus disease outbreak (2014–2016) on tuberculosis surveillance activities by Guinea’s National Tuberculosis Control Program: a time series analysis

**DOI:** 10.1186/s12889-020-09230-2

**Published:** 2020-08-04

**Authors:** Aboubacar Sidiki Magassouba, Boubacar Djelo Diallo, Lansana Mady Camara, Kadiatou Sow, Souleymane Camara, Boubacar Bah, Alpha Oumar Barry, Thierno Hassane Diallo, Aboubacar Camara, Adama Marie Bangoura, Oumou Younoussa Sow

**Affiliations:** 1 National Tuberculosis Control Programme, Conakry, Guinea; 2 Faculty of Health Science and Technology, Gamal Abdel Nasser University of Conakry, Pneumo-Physiology Department, University Hospital, Ignace Deen, Conakry, Guinea; 3 Aix-Marseille University, Marseille, France; 4 Career Tuberculosis Centre, Conakry, Guinea; 5 Pneumo-Physiology Service, Conakry University Hospital, Ignace Deen National Hospital, Conakry, Guinea; 6 National TB Control Program, Conakry, Guinea; 7 Faculty of Health Science and Technology, Gamal Abdel Nasser University of Conakry, Conakry, Guinea

**Keywords:** Tuberculosis, Ebola virus disease (EVD) outbreak, Surveillance system, National Tuberculosis Control Program, Guinea

## Abstract

**Background:**

Most countries in Subsaharan Africa have well-established National Tuberculosis Control Programs with relatively stable routine performances. However, major epidemiological events may result in significant disruptions. In March 2014, the World Health Organization announced the outbreak of Ebola virus disease in Guinea, a country with a high incidence of TB and HIV. Our study aimed to assess the impact of the Ebola virus disease outbreak on TB notification, treatment, and surveillance, using main indicators.

**Methods:**

This is a retrospective cohort study that compared TB trends using surveillance data from the periods before (2011–2013), during (2014–2016), and after (2017–2018) Ebola virus disease outbreak. A time-series analysis was conducted to investigate the linkages between the decline in TB notification and the Ebola virus disease outbreak through cross-correlation. The lag in the cross-correlation test was evaluated using ANCOVA type II delayed variable dependent model. The surveillance system was assessed using TB surveillance standards and benchmarks and vital registration systems recommended by WHO, compared with those of 2015 during the Ebola virus disease.

**Results:**

The rate of reporting of TB declined from 120 cases per 100,000 in 2011 to 100 cases per 100,000 in 2014, at the peak of the Ebola virus disease outbreak. The time-series cross-correlation test of all notified cases of TB and Ebola showed a significant lag of − 0.4 (40%), reflecting a drop in the rate of notification (F-value = 5.7 [95% CI: 0.2–21.3]). The Ebola virus disease had no negative impact on patient treatment outcomes (F-value = 1.3 [95% CI: 0.0–8.8]). Regarding the surveillance system, five out of 13 WHO standards and benchmarks were met following their evaluation in 2019, after the Ebola virus disease outbreak, compared to three in 2015.

**Conclusion:**

Major epidemics such as the Ebola virus disease outbreak may have a significant impact on well-established TB control programs as shown in the example of Guinea. Sudden disruptions of routine performance may lead programs to improve their surveillance system. The experience acquired in the fight against EVD and the investments made should make it possible to prepare the health system in a coherent manner for the other probable episodes.

## Background

Although curable in the majority of cases, tuberculosis (TB) is the leading cause of death due to infectious diseases worldwide [1]. The Ministry of Health of the Republic of Guinea prioritized tuberculosis, among diseases under surveillance, by establishing the National Tuberculosis Control Program (NTP) in 1990 [2]. In 2014, the Guinean government aligned its TB control policy with the WHO strategy on tuberculosis, developed in connection with the Sustainable Development Goals. Efforts were made to align indicators and targets to the context of a developing country [3].

The Ebola virus disease (EVD) outbreak, which occurred in Guinea between 2014 and 2016, had an adverse impact on all health activities. It has been the most severe and long-lasting outbreak that occurred in West African countries (Guinea, Sierra Leone, and Liberia). The outbreak in West Africa in 2014–2016 is unprecedented in terms of the number of cases and deaths [4, 5]. Guinea reported a total of 3818 cases of Ebola, with 2543 deaths nationwide during the outbreak. In addition to its devastating health effects, EVD outbreak also had significant socio-economic impacts in Guinea, Liberia, and Sierra Leone [6]. According to World Bank forecasts in 2016 [7], the overall impact of the Ebola crisis on Guinea, Liberia, and Sierra Leone are estimated at $ 2.8 billion ($ 600 million for Guinea, $ 300 million for Liberia and $ 1.9 billion for Sierra Leone).

The Guinean health system was severely hit by EVD outbreak, primarily due to lack of infrastructure and skilled workers [8]. With a population of over 11 million, the country has only one doctor and one nurse per 10,000 people, three times less than Nigeria, eight times less than South Africa, and 84 times less than Cuba [9]. This weak health care providers to population ratio further deteriorated due to the high rate of Ebola infections and deaths among health workers (192 Ebola infections, including 86 deaths).

Based on these findings, it is hypothesized that EVD outbreak impacted the TB surveillance system in Guinea with an increase in TB-related morbidity and mortality. Our study sets out to assess the impact of EVD outbreak on Guinea’s NTP surveillance activities by analyzing trends in selected indicators before, during, and after the outbreak.

## Methods

### Location and period of study

**Fig. 1:**
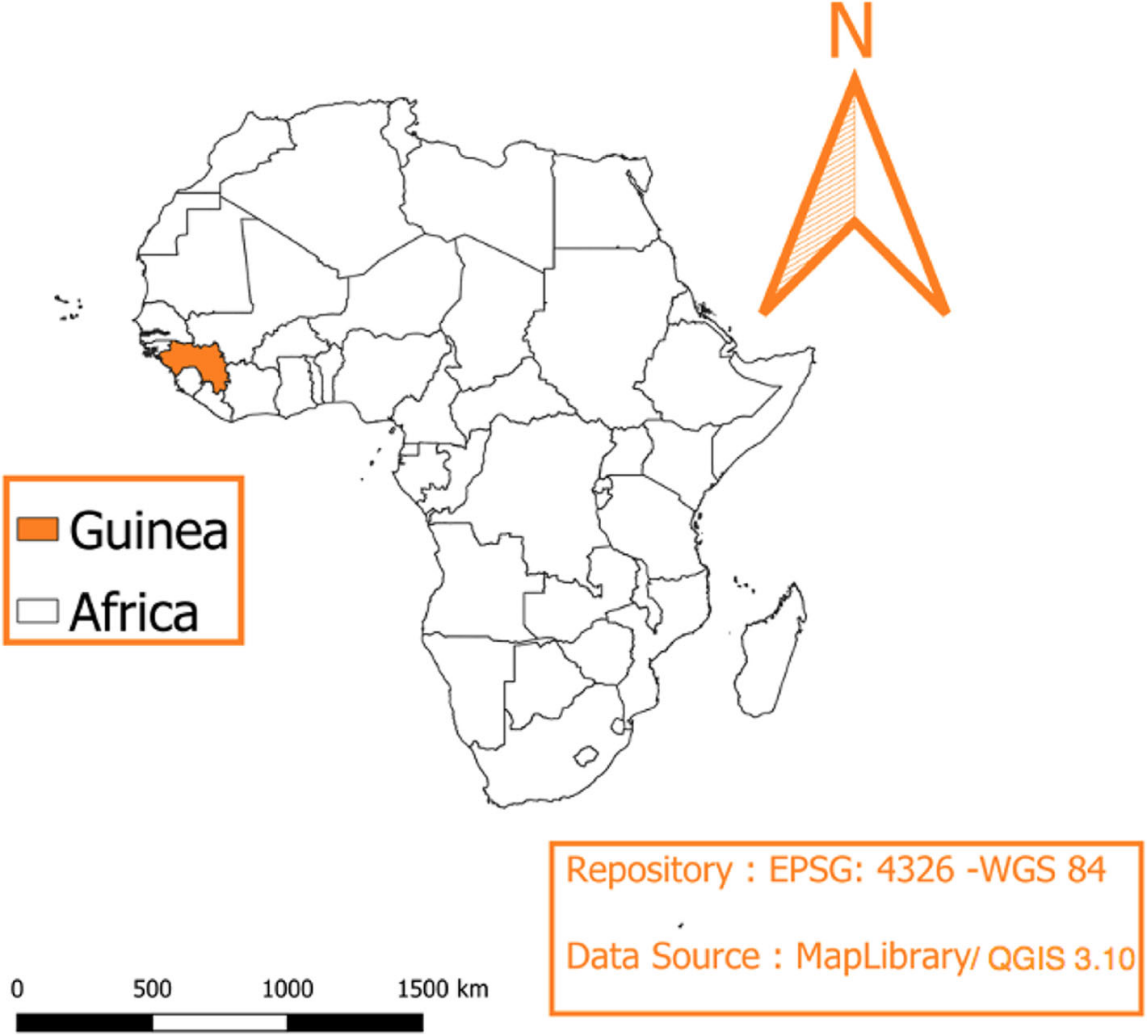
Geographical location of the place of study

The study was carried out at the Guinea NTP (Fig. 1), from February 2019 to June 2019. This Program is under the responsibility of the National Directorate for Major Endemics, the main body in charge of TB epidemiological surveillance, notification, and treatment, under the authority of the Ministry of Health.

### Study design and population

A retrospective cohort study was conducted with a view to determining trends, over time, in TB notification and treatment outcomes for the periods before (2011–2013), during (2014–2016), and after (2017–2018) EVD outbreak. The state of the surveillance system was assessed in comparison to the 2015 assessment during the EVD outbreak in order to analyze trends after EVD outbreak using certain indicators.

### Data used

We included all tuberculosis surveillance data validated by the PNLAT (annual data are validated in the first quarter of each year) and all complete surveillance data validated for the Ebola epidemic. The TB surveillance data used in this study is extracted from the National Health Information System (DHIS2) which contains a module on the collection and analysis of TB data from 2011 to 2018, validated and published by the NTP. Cases of TB were collected and reported quarterly by diagnosis and treatment centers in all health districts since 2016. Aggregated historical surveillance data (2009–2015) from NTP were imported into this system; data entry is ongoing throughout the country on a quarterly basis.

Population estimates were obtained from the National Statistics Institute (NSI), which conducted a general population census in 2014, including forecasts up to 2020. The EVD outbreak surveillance data was obtained from the National Health Security Agency (NHSA), which monitors epidemics in the country. All surveillance data for TB and EVD in Guinea are fully anonymized and available for free access on the WHO website.

### Operational definition of variables

The indicators analyzed in this study are generated from the data collected from the completed quarterly reports of diagnosis and treatment centers. These indicators are consistent with the WHO revised framework for TB reporting, published in 2013 [10]. Two indicators were targeted; TB case reporting indicator and the treatment outcome indicator as described below:

- Tuberculosis notification rate: number of reported TB cases per 100,000 population. This comprises cases that are either bacteriologically confirmed or clinically diagnosed.
- Therapeutic success rate: Percentage of reported TB patients who have been successfully treated.
- Ebola notification rate: number of reported Ebola cases per 10,000 population.

### Statistical analysis plan

A descriptive analysis was conducted for each of the three periods in order to evaluate TB notification rates per 100,000 inhabitants. The 13 WHO standards and benchmarks for TB surveillance and vital registration systems [11] was used to assess the status of the current surveillance system compared to the 2015 assessment during the EVD outbreak.

A time series analysis was conducted to assess the effect of EVD outbreak on the notification and treatment of TB cases by quarter. To that effect, the auto-correlation test was used to examine the significance of the shifts observed in each time series separately and the cross-correlation coefficient to explore the relationship between the time series of Ebola and those of TB.

Stationarity is necessary for the research of cross-correlation between two time-series; it is defined by a constant average and equal variance at any time and can be obtained by diversion or differentiation. The Dickey-Fuller test was used to check the stationarity of our time series; then, the seasonal series was transformed into a stationary series by differentiation [12]. Differentiation is the sequential subtraction of the *xt* value of *xt + 1* from a time series to determine subsequent changes over time [13] . This technique helps to remove spurious correlations based on time dependencies between adjacent values in the input time series and removes these influences from the output time series [14]. To confirm and elucidate the correlations observed between times series in the cross-correlation test, an analysis of interrupted time series (ITS) was performed using the type II Sum Squares ANCOVA lagged dependent variable model [15]. A default boot template, which executes 1000 replications of the primary model with randomly drawn samples to drive the 95% CI bootstrap was included. An adjusted F-value (10% suppression) is reported, and a *p*-value initiated is derived from it. This model is adjusted while estimating the mean difference of dependent variables (TB cases notified) between interrupted periods (EVD, 2014 to 2016) and uninterrupted periods (2011 to 2013 and 2017 to 2018), taking into account the lag of the dependent variable and any other specified co-variate. The significance was defined as a value of p less than 0.05. Data was analyzed using the DHIS2, Excel, and R 3.5.1 software.

## Results

**Fig. 2:**
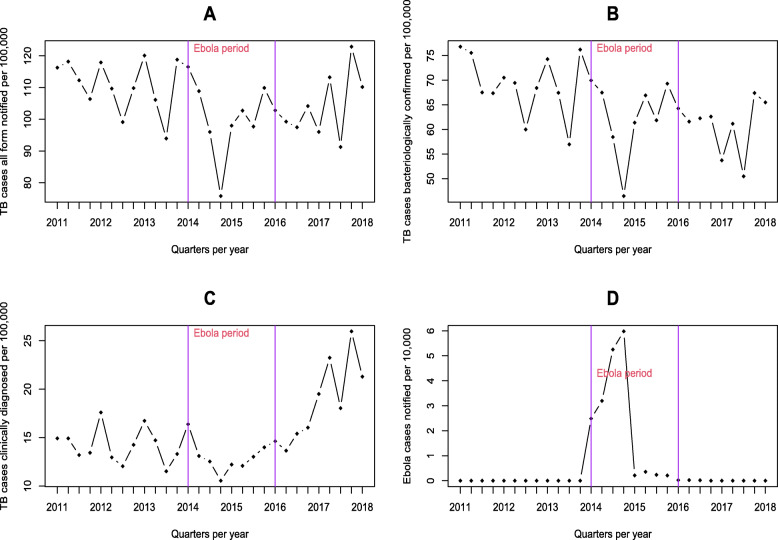
Evolution of the reporting rates of incident cases of (**a**) TB all forms, (**b**) TB bacteriologically confirmed, (**c**) TB clinical diagnosis, and (**d**) Ebola.

On average, 3090 ± 410 cases of TB were reported every quarter in Guinea, with a success rate of 83% ± 0.7 cases, for all the periods considered under our series. The number of TB cases reported varied considerably during EVD outbreak, for all forms of TB, including those clinically diagnosed (468.4 ± 160.1 cases) or bacteriologically confirmed (1872.9 ± 213.1 cases) on average (Table 1). The NTP TB case notification rate fell from 120 cases per 100,000 population in 2011 to 100 cases per 100,000 population in 2014; but resurged from 2015 to 2018 (Fig. 2a). From 2012 to 2013, the trends were slightly downward, but higher than in 2014. After this year, notification of new cases and relapses for all forms of TB started to increase. Likewise, the number of new clinically diagnosed and bacteriologically confirmed TB cases and relapses has resumed the upward trend. This upward trend recorded just after the historic decline in 2014 is very obvious and continues to increase each year (Fig. 2b and Fig. 2c).

**Table 1:** *3Descriptive statistics of TB and Ebola time series*

Variables	Mean (sd)
TB cases all form notified	3086.9 (407.9)
TB cases bacteriologically confirmed	1872.9 (213.1)
TB cases clinically diagnosed	468.4 (160.1)
Ebola cases notified	119.3 (333.7)
Therapeutic success rate	83.9 (4.0)

*Sd* standard deviation, *p < 0.05

From 2011 to 2014, notification rates for all forms of TB cases (F-value = 5.7 [95% CI: 0.2–21.3] and *p*-value = 0.024), bacteriologically confirmed and clinically diagnosed pulmonary cases showed negative variations, that is, a decrease in the number of cases detected each year with a peak of − 26 for bacteriologically confirmed cases in 2014. As of 2015, annual variations ranging from 7% for new clinically diagnosed TB cases to 17% for bacteriologically confirmed TB cases (Fig. 2b).

Analysis of the NTP notifications time series notably shows a larger gap between 2014 and 2015 (F-value = 5.7 [95% CI: 0.2–21.3] and *p*-value = 0.03 for EVD periods). From 2011 to 2018, cascades are observed over the years for all forms of reported TB cases, ranging from of 2000 to 4000 cases per quarter, with year-on-year variations. The periods between 2014 and 2015 reported the fewest cases (2000) compared to other years, where at least 2500 cases were reported.

Concerning Ebola, the number of cases gradually increased, going from 390 cumulative cases during the first two quarters of 2014 to almost 2800 cases (6 cases per 10,000 inhabitants) during the last two quarters of the same year. On the other hand, the NTP barely reported 4500 cases of tuberculosis declared during this period, compared with 5900 during the first two quarters (Fig. 2d). Between July 2014 and January 2015, fewer TB cases (2200 or 94 per 100,000 population) were reported in January and July 2014, increasing to more than 3000 cases (117 cases per 1000 cases) in the first quarter of 2015 as EVD began to decline. Looking at all forms of TB, cases exceed 2500 (96 cases per 1000 inhabitants) per quarter, but the trends were downward in 2014.

The incidence of EVD rapidly changed—increasing and then decreasing, with the most significant proportion occurring before 2014 (more than 500 cases). TB case notification decreased by 1500 cases between 2014 and 2015 before fluctuating the following year positively and then stabilizing until 2016.

**Fig. 3:**
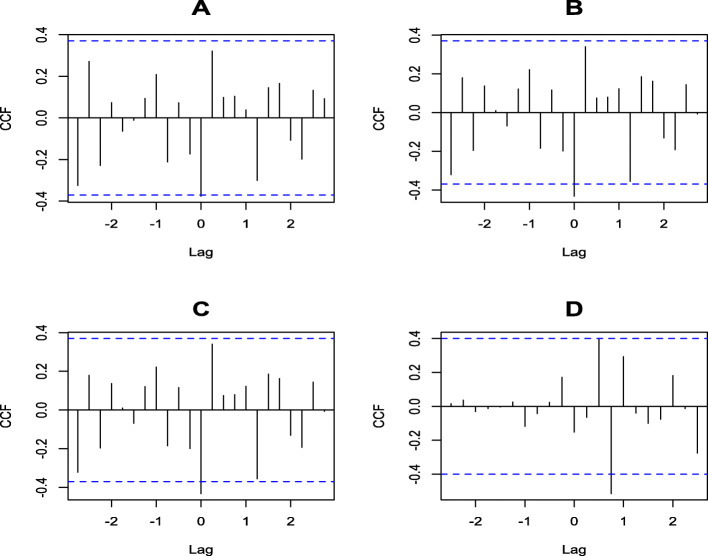
Cross-correlation test for time series of (**a**) Ebola and all forms of TB, (**b**) Ebola and bacteriologically confirmed TB, (**c**) Ebola and clinically diagnosed TB, and (**d**) Ebola and therapeutic success rate

The cross-correlation test between the time series of TB and EVD (Table 2 and Fig. 3a) shows a significant lag of − 0.4 (40%) for all forms of TB, corresponding to the sharp decline in the notification of TB cases observed at the peak of the EVD outbreak in 2014. The ANCOVA model (Table 3) confirms this shift with a *p*-value of the adjusted value of the F-value < 0.01. Although the offsets are observed for the other forms of TB by looking at them separately, these offsets are not significant according to the regression model of the interrupted time series (adjusted *p*-value of F-value> 0.05) despite the seasonal adjustment of the time series (Table 3). The number of reported cases of TB, all forms combined, increased from an average of 2909 cases per 100,000 before the EVD outbreak to 3500 cases per quarter after the EVD outbreak (Table 4), representing a 21% increase (F-values = 11.4 95% CI [0.3–44.1 and *p*-Value = 0.002]). This increase is quite remarkable considering the therapeutic success rate, which averaged 82% before the EVD outbreak and 89% after the outbreak (F-value = 21.9 95% CI [8.9–47.5]) and *p-*value < 0.001).

**Table 2:** Results of the time series seasonal adjustment test4

Variables	Dickey-Fuller Test	Lag order	***p***-value
TB cases all form notified	−3.5	3	0.04*
TB cases bacteriologically confirmed	−3.4	3	0.05*
TB cases clinically diagnosed	−3.8	3	0.03*
Ebola cases notified	−2.4	2	0.02*
Therapeutic-success-rate	0.1	3	0.05*

**Table 3:** *ITS analysis model results on TB surveillance during the EVD outbreak*

Parameters	Sum Sq	Df	Mean F-value (95% IC)	Pr(>F)
TB cases all form notified (TBAF)				
EVD Period	467.0	1	5.7 (0.2–21.3)	0.033
lag_TBAF	24.2	1	0.8 (0.0–6.2)	0.60
Bootstrapped F-values			5.7 (0.2–21.3)	0.024*
TB cases bacteriologically confirmed (TBBC)				
EVD Period	39.7	1	1.4 (0.0–8.6)	0.342
lag_TBBC	102.2	1	2.9 (0.0–13.5)	0.132
Bootstrapped F-values			1.4 (0.0–8.6)	0.247
Lower CI Mean F-values				
TB cases clinically diagnosed (TBCD)				
EVD Period	8.6	1	1.0	0.321
lag_TBCD	176.0	1	20.8	0.0001
Bootstrapped F-values			1.2 (0.0–6.7)	0.275
Therapeutic Success Rate (TSR)				
EVD Period	9.0	1	1.2 (0.0–8.2)	0.417
lag_TSR	89.7	1	10.9 (0.3–36.8)	0.015
Bootstrapped F-values			1.3 (0.0–8.8)	0.258

*Sum Sq* Sum of Squares, *DF* Degrees of freedom, *F* Fisher’s test, *p < 0.05

**Table 4:** ITS analysis model results on TB surveillance after the EVD outbreak

Parameters	Sum Sq	Df	Mean F-value (95% IC)	Pr(>F)
TB cases all form notified (TBAF)				
Post EVD Period	33.5	1	11.4 (0.3–44.1)	0.005
lag_ TBAF	28.8	1	8.8 (0.5–31)	0.008
Bootstrapped F-values			11.4 (0.3–44.1)	0.002**
TB cases clinically diagnosed (TBCD)				
Post EVD Period	97.5	1	21.1 (4.4–64.1)	0.0002
lag_TBCD	5.6	1	1.9 (0.0–14.2)	0.31
Bootstrapped F-values			21.1 (4.4–64.1)	< 0,001**
TB cases bacteriologically confirmed (TBBC)				
Post EVD Period	46.3	1	1.8 (0.0–12.3)	0.30
lag_TBBC	65.3	1	2.3 (0.0–11.3)	0.22
Bootstrapped F-values			1.8 (0.0–12.3)	0.1907
Therapeutic Success Rate (TSR)				
Post EVD Period	156.0	1	21.9 (8.9–47.5)	< 0,001
lag_TSR	1.4	1	1.1 (0.0–9.9)	0.678
Bootstrapped F-values			21.9 (8.9–47.5)	< 0,001**

*Sum Sq* Sum of Squares, *DF* Degrees of freedom, *F* Fisher’s test, **p < 0.01

Regarding the TB surveillance system, of the 13 standards and criteria developed by WHO, five were met by the NTP in 2019, compared to only three in 2015 (Table 5). This means that the surveillance system deserves targeted, long-term action to meet the challenge of screening and monitoring patients on treatment.

**Table 5:** Summary of 13 surveillance standards (comparison of epidemiological reviews 2015 and 2019)

Standard		2015	2019	Comment
**Quality of TB surveillance data**				
**B1.1**	Case definitions are in line with WHO guidelines	**+**	**+**	Adoption of WHO definitions (2013)
**B1.2**	The TB surveillance system is designed to account for a minimum of variables for reported TB cases	**±**	**+**	Adoption of WHO definitions (2013)
**B1.3**	All data to be submitted periodically has been received and processed at the national level	**x**	**+**	100% of reports received in DHIS2 (2018)
**B1.4**	The data presented in the quarterly reports are accurate, comprehensive, consistent internally	**x**	**+**	Quarterly reports show discrepancies in places that are corrected during supervision
**B1.5**	The data contained in the national database are accurate, comprehensive, consistent internally and without duplicates	**NA**	**NA**	Not applicable for a paper-based system
**B1.6**	Tuberculosis surveillance data are externally consistent (5–15% of cases are children)	**+**	**+**	6.5% of our patients were in 2018
**B1.7**	The number of TB cases reported is consistent internally	**±**	**±**	Quarterly reports show discrepancies in places that are corrected during supervision.
**B1.8**	All cases of tuberculosis diagnosed are reported	**x**	**x**	Tuberculosis is under surveillance and is considered a priority for the Ministry of Health. There is no ministerial decree making TB disease notification mandatory.
**B1.9**	People have good access to health care	**x**	**x**	- The under-five mortality rate (probability of dying before the age of 5 per 1000 live births) is 86 per 1000 in 2017 - 54% of health spending was direct payments in 2015
**Quality and coverage of civil state facts**				
**B1.10**	The civil registration system is of excellent quality and provides broad national coverage	**x**	**x**	Guinea has a civil registration system, and there is a register of deaths at the morgue level where deaths are recorded. The causes of death are not recorded.
**Surveillance of Multidrug-resistant Tuberculosis**				
**B2.1**	Surveillance data provide a direct measure of multi-drug resistant TB cases among new cases	**x**	**x**	In our context, not all patients are systematically tested. The Xpert MTB test is indicated for a number of cases. No drug resistance studies have yet been conducted in Guinea
**Tuberculosis/HIV surveillance**				
**B2.2**	Surveillance data provide a direct measure of HIV prevalence in TB patients			The HIV test coverage was 90% in 2018
**Tuberculosis surveillance in children**				
**B2.3**	Surveillance data for children reported to be TB (aged between 0 and 14 by definition) are reliable and accurate, AND all cases of childhood tuberculosis diagnosed are reported	x	x	The ratio of (0–4:5–14 years) patients was 0.38 in 2018

Legend: **x** ‘Standard not reached’; **±** ‘partially reached standard’; **+** ‘Achieved Standard’; NA ‘Not applicable’

## Discussion

The WHO Global Tuberculosis Program estimates the number of tuberculosis incident cases each year for most countries. The number of cases notified by the PNT of Guinea remains continuously lower than the estimates (more than 7000 cases of tuberculosis miss on average each year). This reporting gap widened considerably during the EVD epidemic, among other things, because some TB treatment centers were transformed into health centers for Ebola patients, which weakened the provision of TB services in several places.

A recent systematic review of the linkages between the Ebola epidemic in West Africa and the health systems in Guinea, Liberia, and Sierra Leone [16] revealed the poor performance of health facilities, partly because of the lack of health personnel during the epidemic, inadequate funding for health, weak monitoring, and communication. A study in Sierra Leone [17] also reported a discrepancy in the relationship between the health system and communities during the EVD outbreak, resulting in very few people resorting to health facilities.

According to our study, the rate of NTP reported TB cases declined from 120 cases per 100,000 population in 2011 to 100 per 100,000 population in 2014, when the number cases of Ebola was at its peak. Similar trends were observed in a study on the impact of Ebola on TB screening and treatment outcomes in Liberia. This study reveals that for all forms of TB, by category and by age group, there was a significant decline in the number of TB cases reported during the last two quarters of 2014 [18]. Rashid et al. also indicated that the EVD outbreak in West Africa had a significant impact on all sectors of the health system, in particular TB control services, resulting in increased tuberculosis transmission, TB-related morbidity, and mortality and weaker adherence to TB treatment [19]. The drop in the reported number of TB cases during the EVD outbreak may also be due to socio-demographic and behavioral factors related to the epidemic. Zachariah et al. cited the death of health workers, the temporary or permanent closure of health facilities, and the inherent fear of contracting EVD or being stigmatized as a demographic factor, all of which has had an impact on TB control [20].

Despite the negative impact of the EVD epidemic on the diagnosis and reporting of tuberculosis cases, the therapeutic success rate remained stable above 80%, with a slight increase after the epidemic. This confirms that diagnosed TB cases were properly managed during the EVD outbreak in Guinea, as attested by several other studies [16, 19, 21], including one in Guinea [18], which registered a higher success rate during the EVD outbreak.

Our data shows that the rate of reporting of new cases and relapses, for all forms of TB, began to improve immediately after deteriorating in 2014. This post-Ebola trend may be due to positive post-epidemic effects, such as improved diagnostic capacities, notably new GeneXpert devices reassigned for TB screening, the opening of new treatment sites, and staff training. Along with these improvements, the TB system score in Guinea improved from 2015 to 2019. Health services improvements after the outbreak were similarly reported in a study on the public health impact of the 2014–2015 EVD outbreak in West Africa [8]. It points out that despite the adverse effects on public health and other sectors, the EVD outbreak brought about many other opportunities for West African. It prompted Guinea to increase health expenditure, recruit an additional 2950 health workers, and begin to prioritize community participation in addressing public health risks [22].

The decline in the number of TB cases reported may be attributable to random practices where statistical tests are not available. Cross-correlation tests between the Ebola virus disease outbreak and TB time series confirmed that the observed decline was statistically significant with offsets beyond the confidence intervals of the cross-correlation curve. The TB cases notification dropped by approximately 1500 cases in 2015 before rising the following year and continuing to increase until the end of the Ebola virus disease outbreak in 2016.

## Conclusion

Our study shows a significant decline in the notification rate of all forms of tuberculosis between 2014 and 2016, during the Ebola virus disease outbreak, regardless of diagnosis method or patient category. As evidenced by cross-correlation analysis and ANCOVA model results, this is attributable to the Ebola virus disease epidemic, which disrupted the entire health system. The outbreak did not affect treatment outcomes for patients who were followed up during the same period. Several investments have been made to keep health services open and also to improve the health system, which is very fragile to be able to cope with possible epidemics.

## Data Availability

Data and R scripts used in the analysis are available on request from the authors.

## Abbreviations

DHIS2: District Health Information System versions 2
EVD: Ebola Virus Disease
HIV: Human Immunodeficiency Virus
ITS: Interrupted Time Series
NHSA: National Health Security Agency
NSI: National Statistical Institute
NTP: National Tuberculosis Control Program
TB: Tuberculosis
WHO: World Health Organization.
